# Data supporting polymerization of anti-fouling polymer brushes polymerized on the pore walls of porous aluminium and titanium oxides

**DOI:** 10.1016/j.dib.2019.103702

**Published:** 2019-02-02

**Authors:** Ekram Wassel, Martha Es-Souni, Ayoub Laghrissi, Artjom Roth, Matthias Dietze, Mohammed Es-Souni

**Affiliations:** a Institute for Materials & Surface Technology (IMST), University of Applied Sciences Kiel, Germany; b Clinic of Dentistry, Christian-Albrecht University, Kiel, Germany

## Abstract

The data presented in this article affords insight into the fabrication and ensuing microstructure of the supported porous anodic aluminum oxide (AAO) and TiO_2_-nanotubes (NT) films that are used for the subsequent grafting of antifouling poly(oligo ethyleneglycol) methylether methacrylate (POEGMA) and poly acrylamide (PAAm) brushes. The experimental procedure for the grafting of POEGMA and PAAm via atom transfer radical polymerization (ATRP) is described in Wassel et al. (2019) https://doi.org/10.1016/j.matdes.2018.107542 [1]. The FTIR spectra of the porous oxides before and after attachment of (3-Aminopropyl)trimethoxysilane (APTMS) are presented. Microscopic images of thick POEGMA films and PAAm on AAO are displayed, and an FTIR spectrum of AAO/PAAm is shown. An EDX mapping of carbon is shown on an AAO/POEGMA sample. The adsorption behavior of Fluorescein isothiocyanate (FITC) marked bovine serum albumin (BSA) on patterned porous TiO_2_-NT films is documented. Finally microscopic images are presented to compare the scratch resistance behavior of pristine porous films with those functionalized with POEGMA.

**Specifications table**

**Table:** 

Subject area	*Chemistry*
More specific subject area	*Materials Science*
Type of data	*Image (SEM, fluorescence microscope, optical microscope, contact angle), graph (IR-spectrum, EDX spectra)*
How data was acquired	*The components of the AAO surface covered with PAAm were investigated using ATR-FTIR. The raw data were transferred to Origin program and analyzed.*
	*The topographical characterization of the pristine and functionalized AAO and TiO*_*2*_*surfaces with polymer brushes were investigated using SEM. The SEM images were analyzed.*
	*The wettability of the AAO and TiO*_*2*_*surfaces were investigated before and after polymerization using contact angle.*
	*The antifouling property of the structured TiO*_*2*_*surfaces was examined using fluorescence Microscope.*
Data format	*Analyzed, Raw*
Experimental factors	*AAO pores were fabricated by electrochemical anodization of deposited Titan//Gold/Aluminium film on glass and Si substrates, while the production of TiO*_*2*_*-nanotubes took place by electrochemical anodization of pure titanium substrates.*
	*For synthesis of POEGMA brushes, BiPy (1.2480 g), 12 mL of deionized water and methanol (1:4), purified OEGMA (19.08 mL) and CuBr (0.5720 g) and for fabrication of PAAm brushes PMDETA, (0.28 mL), 20 mL of deionized water and methanol (3:7), AAm (2.00 g) and CuBr (0.064 g) were used. The AAO and TiO*_*2*_*surfaces and pores were first modified via chemical vapour deposition (CVD) with APTMS that possess the aminofunctional groups acting as a coupling units for the attachment of the atom transfer radical polymerization (ATRP) initiator (SI-ATRP) for the synthesis of OEGMA and AAm brushes.*
	*In order to prove POEGMA brushes as antifouling system, a mechanical mask was placed on the TiO*_*2*_*-NT surface using a permanent magnet to produce a structured surface with APTMS attachment in the gaps. Therefore ATRP reaction was only succeeded in the gaps. The structured sample was incubated in aqueous solution of FITC-labelled BSA (3 mg/mL) for 2 hours at 37 °C, rinsed with water, dried with nitrogen gas and investigated using a fluorescence microscope.*
Experimental features	*Different methods show the covalent grafting of POEGMA and AAm brushes on AAO and TiO*_*2*_*surfaces and inside the pores.*
	*Verification of non-fouling property of POEGMA brushes on TiO*_*2*_*-NT was performed using Adsorption of bovine serum albumin (BSA) marked with fluorescein isothiocyanate (FITC).*
Data source location	*Kiel, Germany*
Data accessibility	*Data are available within this article*
Related research article	E. Wassel, Mar. Es-Souni, A. Laghrissi, A. Roth, M. Dietze, M. Es-Souni, Scratch resistant non-fouling surfaces via grafting non-fouling polymers on the pore walls of supported porous oxide structures,Materials and Design 193 (2019) 107542–107551 [1].

**Value of the data**

● These data demonstrate the successful grafting of antifouling poly(oligo ethyleneglycol) methylether methacrylate (POEGMA) and acrylamide (AAm) brushes directly onto the pore walls of supported porous anodized aluminum oxide (AAO), generating robust transparent organic-inorganic anti-adhesive nanocomposite films that can be used for displays and as windows for submerse optical sensors.
● Our data also verify the covalent-grafting of antifouling polymer brushes onto the pore walls of TiO_2_-nanotubes (NT), yielding 3D anti-adhesive surfaces that can be used for Ti-base surgical implants. These porous oxide materials afford a huge surface area.
● The anti-adhesive properties of anchored POEGMA brushes into the patterned 3D porous TiO_2_-NT films were investigated and proved using Fluorescein isothiocyanate (FITC) marked bovine serum albumin (BSA). The data obtained point to the strong potential of our 3D anti-fouling coatings as candidates in the area of health and biomedical research.

## 1 Data

**Fig. 1:**
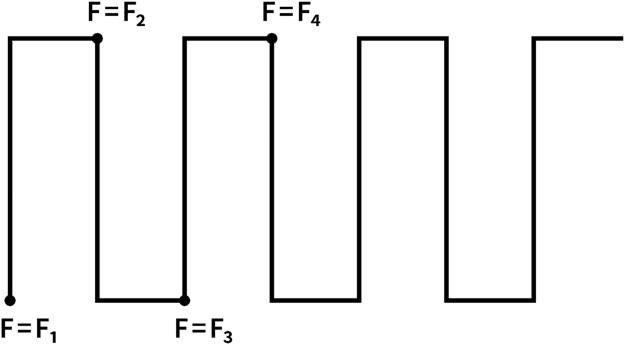
A drawing showing the test procedure for the scratch test.

Data presented in this article displays a drawing showing the test procedure for the scratch test (Fig. 1).

**Fig. 2:**
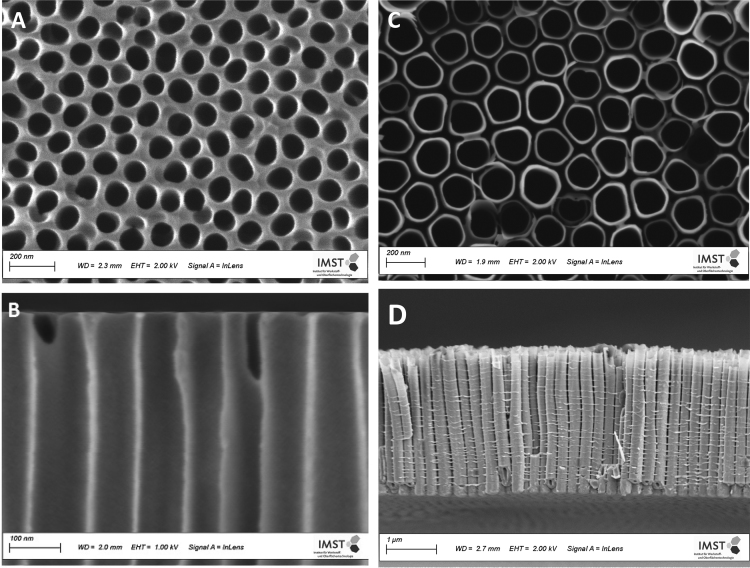
(A) SEM micrograph of unmodified AAO pores (top view), (B) SEM micrograph of uncoated AAO pores (cross section), (C) SEM micrograph of unmodified TiO_2-_NT pores (top view), (D) SEM micrograph of uncoated TiO_2_-NT pores (cross section).

The following data in this document, obtained by various methods of measurement, demonstrate the successful synthesis and grafting of polymer brushes on the surface and in the pores of AAO and TIO_2_-NT.The SEM images show top views and cross sections of pristine AAO pores (Fig. 2(A), (B)) with a mean pore diameter of 80 nm and of unmodified TiO_2-_NT pores with a mean pore diameter of 150 nm (Fig. 2(C), (D)).

**Fig. 3:**
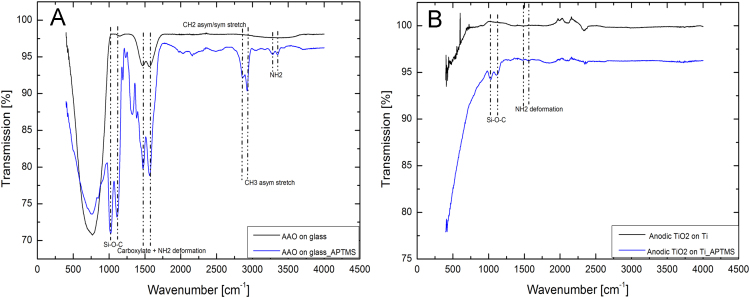
ATR-FTIR spectra of the pristine and aminosilane-functionalized (A) AAO and (B) TiO_2_-NT.

ATR-FTIR spectra illustrated in this paper reveal the pristine and silane-functionalized AAO and TiO_2_ samples (Fig. 3 (A) and (B)). While pristine AAO sample shows vibrations at 1560 and 1460 cm^−1^ which belong to the carboxylate groups that are integrated into the aluminium oxide during anodization in oxalic acid electrolyte, the APTMS-modified AAO sample reveals Si-O-C stretching at 1000–1100 cm^−1^, overlapped NH_2_ deformation with carboxylate groups at 1476–1583 cm^−1^, CH_2_ symmetric/asymmetric stretching at 2863 cm^−1^ and CH_3_ symmetric stretching at 2929 cm^−1^. The strong absorption between 1000 and 500 cm^−1^ pertains to the glass substrate. The same vibrations can also be seen for APTMS-modified TiO_2_-NT.

**Fig. 4:**
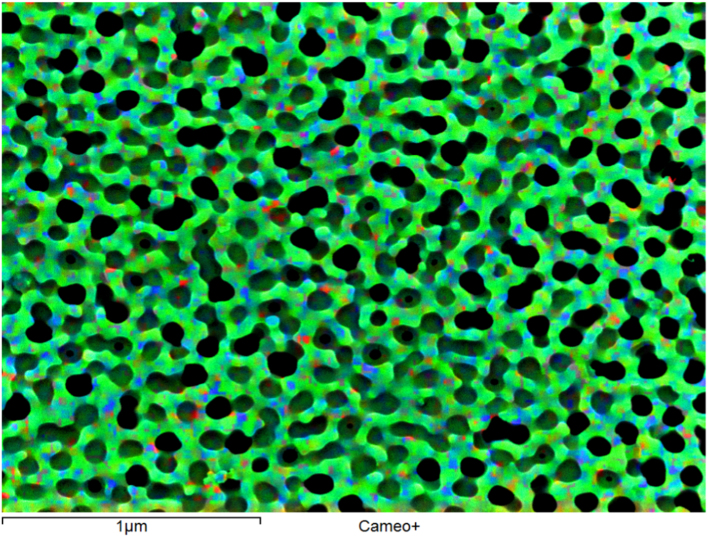
EDX mapping of carbon (green) superimposed on the secondary electron micrograph.

EDX mapping of carbon (green) superimposed on a secondary electron micrograph is displayed in Fig. 4.

**Fig. 5:**
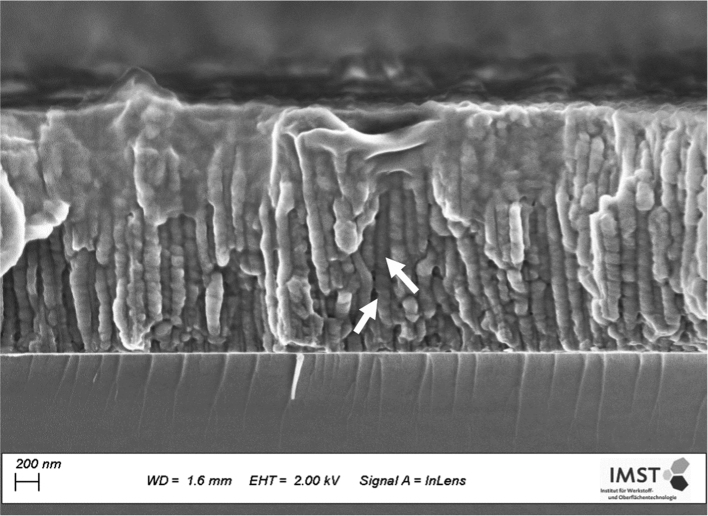
SEM micrograph of AAO pores-cross section, which are fully filled with POEGMA nanofilm (arrows) after 2 h polymerization.

**Fig. 6:**
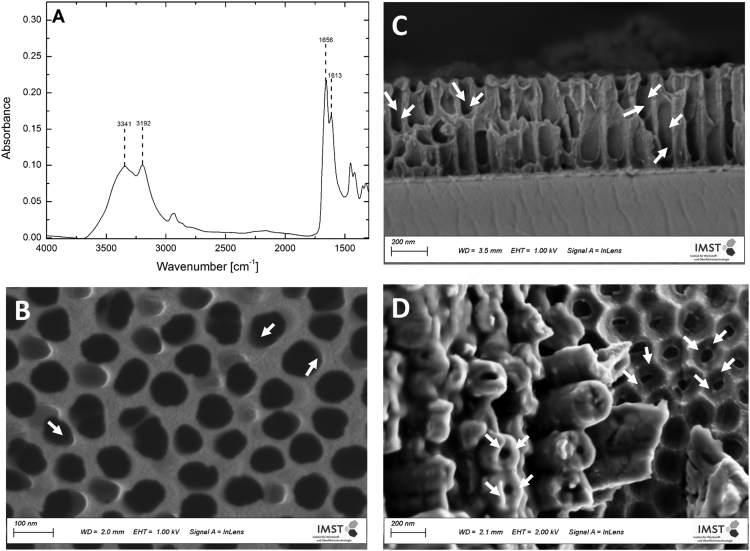
(A) FTIR signals of PAAm brushes at 1656 and 1613 cm^−1^ are assigned to the amide I and amide II vibrations. The broad absorption bands at 3341 and 3192 cm^−1^ are attributed to the stretching vibrations of the amine functionality., (B) is a HR, top-view SEM micrograph showing the PAAm film covering the AAO pores (arrows) (C) SEM micrograph of the cross-section of AAO, confirming that the PAAm nanofilm reaches down to the pore base (D) top view of the porous structure of TiO_2_-NT where the grey shades designated by arrows show the PAAm film.

Not only the obtained SEM micrograph (Fig. 5) proves the coating of AAO pores with POEGMA nanofilm, but also IR spectra (Fig. 6A) and the recorded SEM images of the AAO surface and nano-pores (Fig. 6(B), (C)) as well as TiO_2_ surface (Fig. (D)) demonstrate the similar grafting of PAAm polymer brushes on the surface and inside of the pores.

**Fig. 7:**
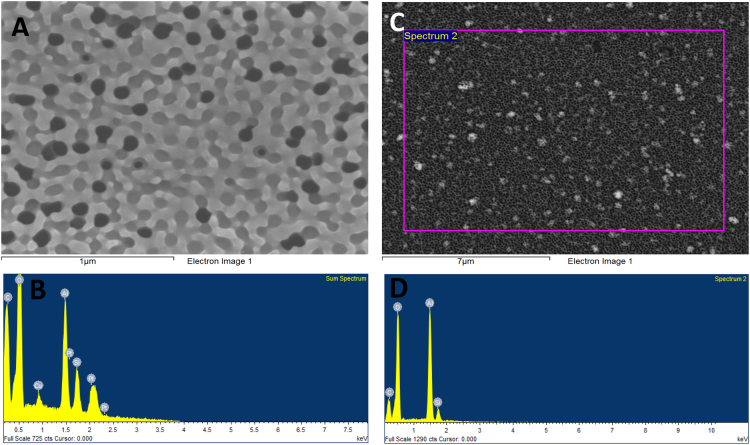
Secondary electron micrographs and EDX analyses of AAO/POEGMA before (A, B) and after (C, D) soaking in water. In B the sample was sputtered with a thin Platinum (Pt)-film for imaging and analysis. Notice that the Cu signal is no more present in D.

Secondary electron micrographs and EDX analyses of AAO/POEGMA before Fig. 7(A) and (B) and after Fig. 7(C) and (D) soaking in water demonstrate the stability of the covalently grafted nanofilms and the instability of non-covalently bounded copper from the copper bromide catalyst that is necessary for the synthesis of polymer brushes.

**Fig. 8:**
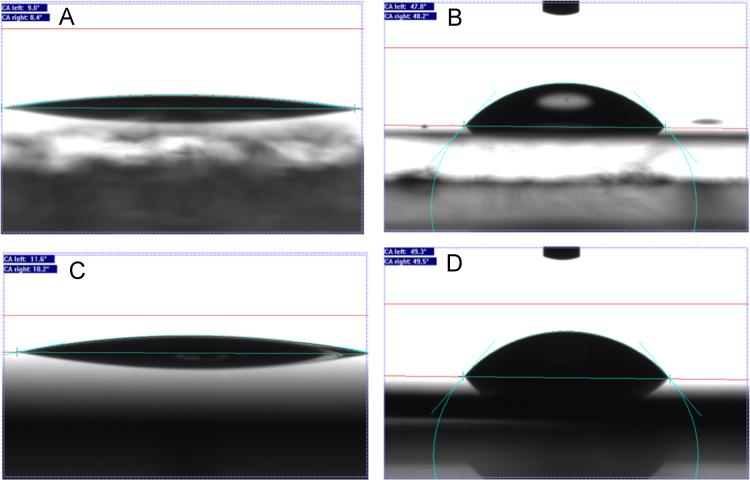
Water contact angle measurements on (A) uncoated AAO substrate, (B) AAO grafted with POEGMA brushes, (C) unmodified TiO_2_-NT surface_,_ (D) TiO_2_-NT grafted with POEGMA brushes.

Different wettability of the AAO and TiO_2_ surfaces before and after grafting of OEGMA brushes (Fig. 8(A)–(D)) are represented using wate contact angle measurements.

**Fig. 9:**
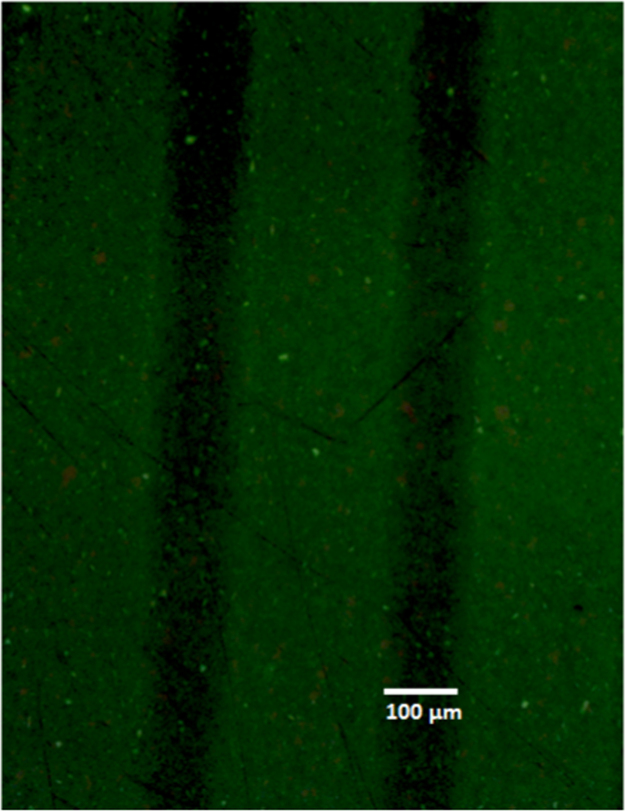
Fluorescence micrograph obtained on a structured surface and subsequently treated with FITC labelled BSA.

The Fluorescence micrograph obtained from a TiO_2_-structured surface and subsequently treated with FITC labelled BSA (Fig. 9) shows green-fluorescent areas representing BSA adsorption on non-grafted regions with POEGMA and dark areas indicating the POEGMA-grafted fields which resist BSA adsorption.

**Fig. 10:**
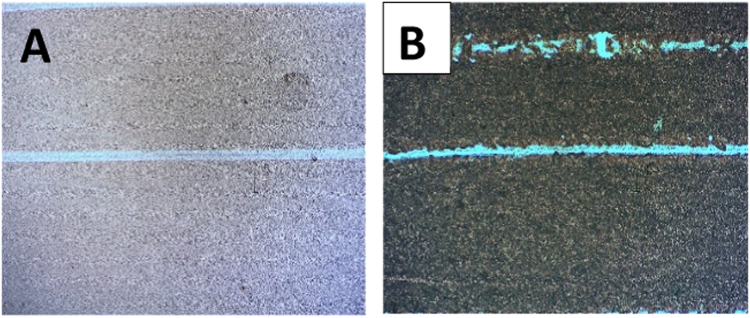
(A) and (B) are optical micrographs of control samples of AAO and TiO_2_-NT, respectively, after a scratch tests at 5 N (AAO) and 4 N (TiO_2_-NT) where in both cases the substrate underneath is revealed.

**Fig. 11:**
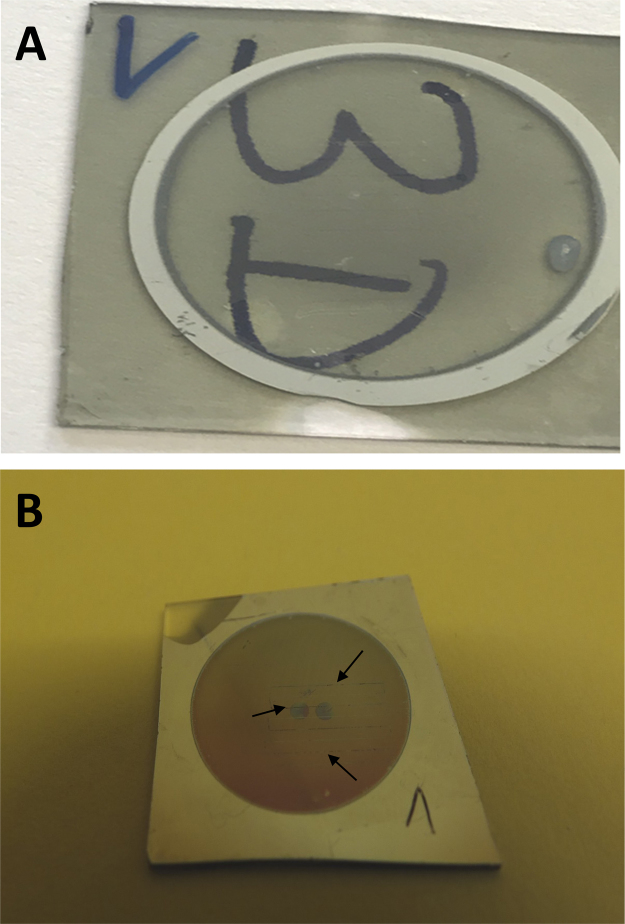
Macrographs of scratched AAO/POEGMA surface (A) in comparison to pristine AAO (control). In B the long scratches may be seen (arrows).

Optical micrographs data illustrate scratch tests of control samples AAO (5 N) (Fig. 10(A)) and TiO_2_-NT (4 N) (Fig. 10 (B)) where in both cases the substrate underneath is revealed. Macrographs of scratched AAO/POEGMA surface. (Fig. 11(A)) in comparison to pristine AAO (control). In Fig. 11(B).

## 2 Experimental design, materials and methods

All experimental design, materials and methods were based on reported paper [1].

### 2.1 Reagents and materials

All used materials were purchased from Sigma-Aldrich, Alfa Aesar, Roth and Kurt J. Lesker.

### 2.2 Fabrication of porous AAO and TiO_2_-NT materials

The AAO film was fabricated via electrochemical anodization of an aluminium film on glass that was pre-coated with a thin titanium/gold heterostructure. Anodization of commercially pure titanium served to make TiO_2_-nanotubes.

### 2.3 Attachment of Aminosilane and ATRP-initiator

The modification of AAO and TiO_2_ surfaces and pores was carried out first by chemical vapor deposition (CVD) with APTMS known as linking moieties for attachment of the atom transfer radical polymerization (ATRP)-initiator for the synthesis of polymer brushes.

### 2.4 Synthesis of polymer brushes

POEGMA brushes were prepared using BiPy (1.2480 g), 12 mL of deionized water and methanol (1:4), purified OEGMA (19.08 mL) and CuBr (0.5720 g), while for synthesis of PAAm brushes PMDETA, (0.28 mL), 20 mL of deionized water and methanol (3:7), AAm (2.00 g) and CuBr (0.064 g) were used.

### 2.5 Adsorption of bovine serum albumin (BSA) marked with fluorescein isothiocyanate (FITC)

Structured samples were incubated in aqueous solution of FITC-labelled BSA (3 mg/mL) for 2 hours at 37 °C, rinsed with water, dried with nitrogen gas and investigated using a fluorescence microscope.

### 2.6 Scratch test

For the scratch test a hardened steel sphere was moved on the surface with a constant load, describing a meander as in Fig. 1. The load is increased to the next value at the end of a meander segment. The displacement rate was 2 mm/s.

### 2.7 Characterization of samples

Attenuated total reflectance Fourier transform infrared spectroscopy (ATR-FTIR), Water contact angle (WCA) and Scanning electron microscopy were used to characterize material chemistry and microstructure, wettability and topography of the pristine - and covered surfaces with polymer brushes.
