# Supplementary material for: Data supporting polymerization of anti-fouling polymer brushes polymerized on the pore walls of porous aluminium and titanium oxides
Source: Data Brief. 2019 Feb 2;23:103702. doi: 10.1016/j.dib.2019.103702 (PMC6383193; doi:10.1016/j.dib.2019.103702)
Supplement: Supplementary file 1 — Transparency document [file mmc1.docx]

Data supporting polymerization of anti-fouling polymer brushes polymerized on the pore walls of porous aluminium and titanium oxides

There is no conflict on interest.

The authors
